# Retrospective Review of Limitations of Care for Inpatients at a Free-Standing, Tertiary Care Children’s Hospital

**DOI:** 10.3390/children5120164

**Published:** 2018-12-10

**Authors:** Christopher J. Plymire, Elissa G. Miller, Meg Frizzola

**Affiliations:** 1 Division of Pediatric Critical Care, Nemours/Alfred I. duPont Hospital for Children, Wilmington, DE 19899, USA; christopher.plymire@nemours.org (C.J.P.); meg.frizzola@nemours.org (M.F.); 2 Division of Palliative Medicine, Nemours/Alfred I. duPont Hospital for Children, Wilmington, DE 19899, USA

**Keywords:** cardiopulmonary resuscitation, resuscitation orders/ethics, palliative care/ethics, humans, hospice care, pediatric intensive care units

## Abstract

Limited studies exist regarding the timing, location, or physicians involved in do-not-resuscitate (DNR) order placement in pediatrics. Prior pediatric studies have noted great variations in practice during end-of-life (EOL) care. This study aims to analyze the timing, location, physician specialties, and demographic factors influencing EOL care in pediatrics. We examined the time preceding and following the implementation of a pediatric palliative care team (PCT) via a 5-year, retrospective chart review of all deceased patients previously admitted to inpatient services. Thirty-five percent (167/471) of the patients in our study died with a DNR order in place. Sixty-two percent of patients died in an ICU following DNR order placement. A difference was noted in DNR order timing between patients on general inpatient units and those discharged to home compared with those in the ICUs (*p* = 0.02). The overall DNR order rate increased following the initiation of the PCT from 30.8% to 39.2% (*p* = 0.05), but no change was noted in the rate of death in the ICUs. Our study demonstrates a variation in the timing of death following DNR order placement when comparing ICUs and general pediatric floors. Following the initiation of the PCT, we saw increased DNR frequency but no change in the interval between a DNR order and death.

## 1. Introduction

The do-not-resuscitate (DNR) order has existed in various forms for more than 30 years [1]. Throughout the past several decades, the importance and role of advanced care planning and limitations of care have continued to expand, yet the DNR order continues to represent an area of concern and confusion for patients and care teams. One recent study noted that for 25% of children reviewed with life-threatening or life-limiting conditions, no discussion was documented regarding the approach to end-of-life care [2].

The initial statement from the American Academy of Pediatrics (AAP) on forgoing life-sustaining treatment was published in 1994 and most recently updated in 2017. The guidelines include recommendations for the open sharing of information, the importance of surrogate decision makers, and the need for “thorough communication among all stakeholders”. The first recommendation proposed by the committee is that, although there is a presumption in favor of sustaining life in most circumstances, forgoing life-sustaining medical treatment is ethically supportable when the burdens of treatment outweigh the benefits to the child [3].

While limited information is available regarding overall hospital and individual unit rates of DNR orders, several recent studies have analyzed physician and nursing attitudes regarding limitations of care. Physicians in the United Kingdom were surveyed on their opinions on end-of-life care. The physician experience levels in the study ranged from junior house staff to senior attending physicians. The questionnaire focused on better understanding physician attitudes and practices surrounding the withholding and discontinuation of life-sustaining treatment. The specific survey questions included the frequency at which the physician held end-of-life discussions, resources used by respondents, and perceived barriers to holding end-of-life discussions. Specific barriers to end-of-life discussion were noted by the survey respondents, including family readiness, family agreement with the overall prognosis, and prognostic uncertainty. The junior medical staff overall were less comfortable holding discussions regarding limitations of care compared with the senior physicians, and only 42% of respondents cited formal training in end-of-life discussions [4].

The discomfort many physicians may feel when faced with discussions surrounding end-of-life care has been echoed by many other members of the healthcare team. A multidisciplinary study by Sanderson et al. [5] investigated the beliefs of both physicians and nurses surrounding DNR order placement for those patients with life-threating illnesses at a large pediatric tertiary care hospital. The study population comprised attending physicians, nurses, advanced practice nurses, and trainees in a variety of hospital settings. The majority of physicians and nurses in the study believed that the patient’s care changed beyond resuscitative measures after a DNR order was in place. Fifty-two percent of respondents noted an increased focus on comfort, including a limitation of diagnostic and therapeutic interventions. The study also noted that the majority of those questioned felt that, in most cases, the DNR discussion was held during a period of acute illness or when death was clearly imminent [5].

Limited studies exist regarding the timing, location, or physicians involved in DNR order placement in pediatrics. One study reviewed 38 cases of withdrawal of life-sustaining treatment in the pediatric intensive care unit (ICU) at Royal Children’s Hospital, Melbourne, Australia. The investigators found that the average time from the first discussion of withdrawal of life-sustaining treatment to the decision to withdraw life-sustaining therapy varied from an immediate decision to 19 days following the initial discussion. While the study reviewed withdrawal of life-sustaining therapy—versus limitation of care or DNR orders—great variations in practice were noted throughout the process of withdrawal of life-sustaining treatment until death [6].

The earliest large-scale study reviewing pediatric in-hospital death was performed by McCallum et al. between 1996 and 1998. The 236-patient study evaluated the care and demographics of children who remained inpatients prior to death. Of the 77 children who remained admitted to the hospital at the time of their death, 83% died in an ICU. Eighty-seven percent of the children in their study had DNR orders in place at the time of death. This study noted that the majority of DNR discussions occurred very late in the course of illness; the time interval from DNR to death averaged <24 h. The study also noted trends that occurred more frequently prior to the popularization of palliative care: patients were rarely told that they were dying, the acuity of care remained very high until the time of death, and end-of-life issues were only discussed very close to the time of death [7].

Prior studies have sought to determine the influence of various cultural and demographic factors on the rate of DNR order placement in terminal conditions. One study investigated the rate of DNR placement for children with incurable solid organ tumors prior to death. Significant differences were noted in the parents’ educational level and monthly family income and amongst parents’ stated religions [8].

The location of the death of children remains an avenue with opportunities for research. A British study showed that, where palliative care services existed, children who were referred to Palliative Care following an intensive care unit stay were more likely to die in the community (home or hospice) [9]. Also, several studies have been completed in specific subpopulations, such as patients with solid organ tumors and leukemia, to identify the rates of hospital discharge prior to death. One recent retrospective review studying children with an oncologic diagnosis from 1999 to 2011 noted that 37% of children with an oncologic diagnosis died at home. Specific racial subgroups were noted to have higher rates of home death, and Caucasian children were the most likely to die at home. Significant variability in the rates of death at home were noted between varying primary oncologic diseases, neoplasms, and leukemia [10].

Our study aims to improve the understanding of factors preceding end-of-life care in pediatrics, notably through patient and physician description, demographics, and the timing and location of DNR discussions and death, comparing the time period before and after the implementation of a pediatric palliative care team.

## 2. Materials and Methods

We identified all patient deaths that occurred in both outpatient and inpatient settings at our institution from January 2009 through August 2014. The study hospital is a 194-bed tertiary care center, including a 24-bed pediatric ICU (PICU), 18-room neonatal ICU (NICU), and 14-bed cardiac ICU (CICU). Our Institutional Review Board approved the retrospective chart-review protocol. We screened for a DNR order for all patients who were documented as deceased. The location and time of death were obtained from the inpatient medical record, death certificate, or home hospice nurse report as documented in the electronic medical record (EMR). All data were stored in a de-identified database, and no family consent was needed.

We included all pediatric patients (0–21 years) previously admitted as an inpatient to the hospital. Demographic data were obtained as available, including self-identified race, ethnicity, language, religion, age, and patient location. The placement of a DNR order was identified through an examination of the inpatient order section and supporting documentation in the patient’s EMR. The DNR order was included if it restricted, at minimum, the administration of CPR; however, many included the restriction of intubation (Do Not Intubate or DNI), extracorporeal membrane oxygenation, or hemodialysis. We recorded the name and specialty of the physician placing the DNR order, as well as the patient location when the DNR order was placed. The study period included a 2-year period prior to the initiation of the palliative care service and a 2-year period following their availability. The presence of a palliative care team consult and whether the palliative care team obtained the DNR status was noted.

The data were analyzed using Excel (Microsoft 2011, Redmond, WA, USA) and SPSS (SPSS version 18; IBM, Chicago, IL, USA). Data are reported as total counts, percentages, and medians. Groups were compared using Mann–Whitney U tests and paired and unpaired *t* tests. A *p* value of less than 0.05 was deemed significant.

## 3. Results

Throughout our study period, there were 471 patient deaths. The study population comprised 167 patients with a DNR order in place prior to death (35.4%). Patient baseline characteristics and demographics are described in Table 1. The average age was 6.5 years, with a range from 3 days to 20 years. The study group was 54% male. The most common self-identified races were Caucasian and African American.

**Figure 1:**
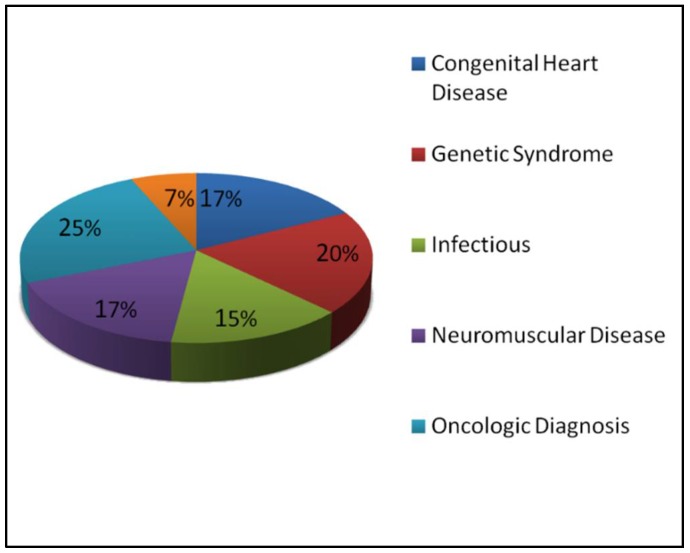
Diagnostic categories of deceased patients during the study period.

The majority of the children included in our study had multiple complex medical problems. The most frequent diagnostic category was a primary oncologic disorder (25%). Congenital heart disease accounted for 17% of patients. Many patients suffered from a combination of neuromuscular disease, genetic syndromes, and chronic respiratory issues, which constituted the majority of the remainder. Seven percent of the patients had an acute event leading to respiratory or cardiovascular arrest at presentation (Figure 1).

**Figure 2:**
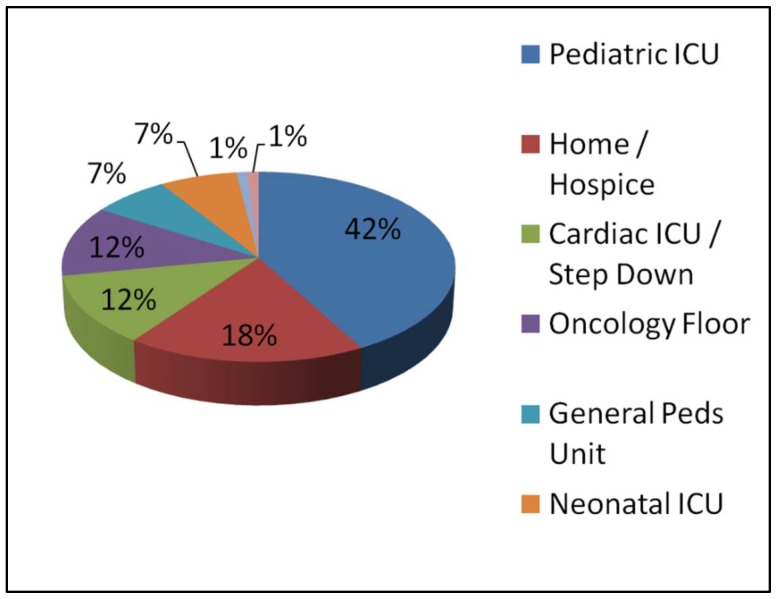
Location of death for patients with a DNR order in place.

Figure 2 demonstrates the location of death for those patients with a DNR order in place. Sixty-two percent of patients died in an intensive care setting, 42% in the PICU. Eighteen percent of patients died either at home, in a chronic nursing care facility, or at an inpatient hospice.

**Figure 3:**
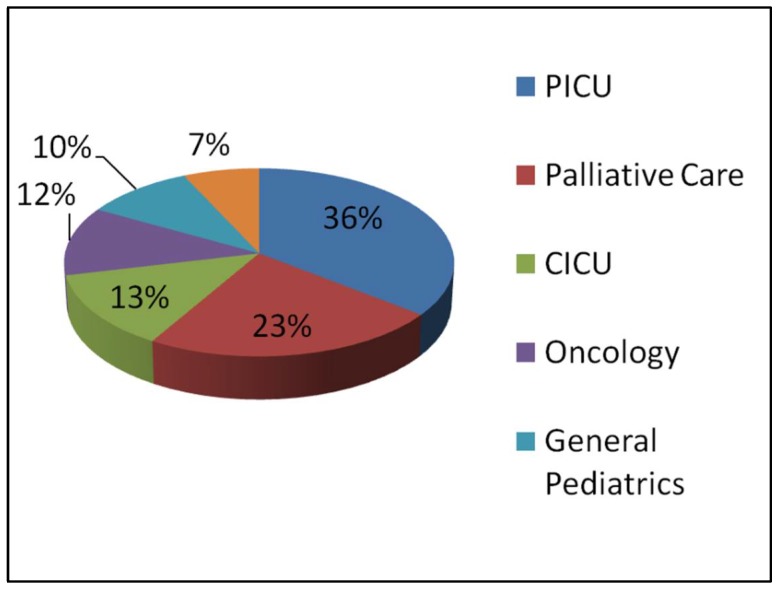
Specialty of physician obtaining the DNR order.

The subspecialty of the physician placing the DNR order is described in Figure 3. The PICU attending wrote the DNR order for 36% of patients, and the palliative care consultant wrote the DNR order for 23% of patients.

**Figure 4:**
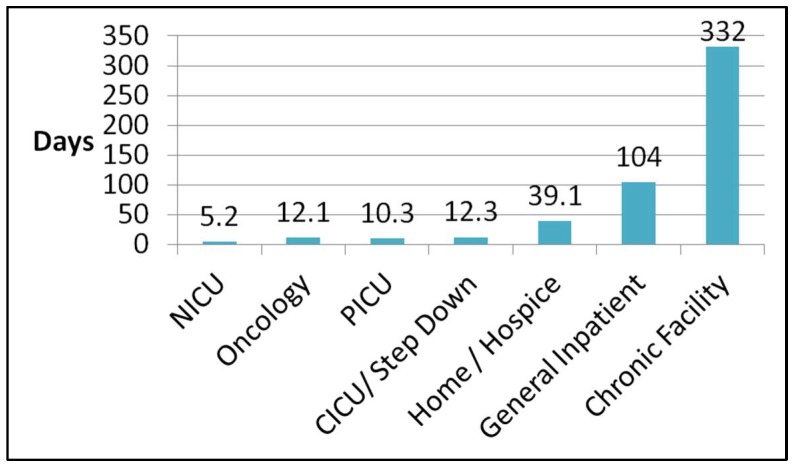
Interval in days between DNR order placement and death. No statistically significant difference (*p* = 0.53) was noted between the neonatal/pediatric/cardiac intensive care units (NICU/PICU/CICU) and the oncologic floor. A statistically significant increase in duration was noted between the general inpatient floor when compared with the ICUs and hematology/oncology unit (*p* < 0.02). Patients who resided at a chronic care facility or nursing home had a significantly longer interval between DNR and death when compared with all other groups (*p* < 0.05).

**Figure 5:**
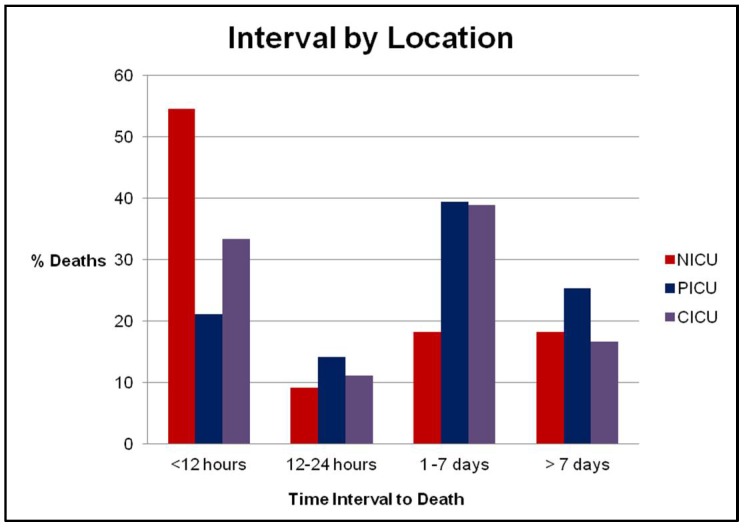
Interval between DNR placement and death in the various ICUs. The majority of deaths in the NICU occurred in the 12 h following DNR order placement (*p* < 0.05).

The time interval between DNR order placement and death was recorded from the most recent DNR order placement in the electronic medical record to the time of death (Figure 4). There was a wide variation in overall intervals to death, from 5.2 days on average in the neonatal ICU to 332 days for patients dying in a chronic facility. No statistical difference was noted for the overall interval between DNR and death in the various intensive care units (NICU/PICU/CICU). Figure 5 demonstrates the interval between DNR placement and death in the various ICUs, stratified by time and location. The majority of patient deaths in the NICU occurred in the 12 h following DNR order placement, as opposed to greater than 12 h in the PICU and CICU (*p* = 0.05).

No significant difference was noted in the time interval from DNR order to death based upon sex, religion, or primary language spoken.

Patients with an oncologic diagnosis on the general inpatient floor had a shorter interval between DNR placement and death (12.1 days) compared with general inpatients (104 days, *p* = 0.03).

The study period included the 2-year period prior to and the 2-year duration following the initiation of a multidisciplinary pediatric palliative care team. Table 2 describes the changes in DNR rates, likelihood of palliative care consultation, and rates of palliative care team DNR placement. The initiation of the pediatric palliative care team was associated with an increase in the rates of hospital-wide DNR order placement, from 30.8% to 39.2% (*p* = 0.05), but not with a change in the interval between DNR placement and death (*p* = 0.28%). For all hospitalized patients with DNR orders in place, the rate of palliative care team consultation was 80%.

## 4. Discussion

In this retrospective study, similar to other published reports, the majority of pediatric deaths occurred in an ICU [11]. This primarily occurred for children with multiple comorbidities and chronic medical conditions following a worsening acute deterioration. Our study demonstrates a widespread average time from DNR placement to death amongst the various patient locations, ranging from 5.2 days in the neonatal ICU to 332 days for those discharged to a chronic care facility. This disparity was not surprising, considering the inherent differences between acute, critical illness and chronic, life-limiting illness.

More interestingly, our study identified that infants in the NICU were statistically more likely to die <12 h following the placement of a DNR order. The discussion of treatment limitations in the NICU has been a recent topic of research. Previous studies have demonstrated that the majority of neonatal intensive care unit deaths follow the withdrawal of life-sustaining treatment. Nearly 50% of infants have therapy discontinued on the basis of a predicted poor prognosis and quality of life [12] The shorter interval observed in our study can likely be attributed to withdrawal of care secondary to the severe congenital malformations, poor prognostic factors, and severe hypoxic encephalopathy noted in many of the neonates.

Our study encompassed an array of patients with a wide spectrum of disease processes and socioeconomic backgrounds. The majority of patients in the study self-identified as either Caucasian or African American, but no significant difference in DNR rates or time to DNR order placement were noted amongst the various demographic groups. Previous studies have reported that various cultural, religious, and ethnic subpopulations were more likely to withhold life-sustaining treatment [8,13]. Our study, despite occurring at a large urban tertiary care center, may have lacked the patient diversity necessary to see a difference in specific subpopulations.

Parent and physician preferences for end-of-life care outside of the hospital setting have recently been explored. One recent survey of families and providers ranked home as the families’ first choice for end-of-life care (70.2%), with a free-standing pediatric hospice as a new and possible option in certain demographic regions but largely unavailable at our institution [14]. In our study population, 18% of the patients died outside of the hospital, either in a chronic residential facility, at home, or in a facility-based hospice.

The palliative care team was formed during our study period. The team comprises a multidisciplinary group of attending physicians, an advanced practice nurse, a social worker, a child life specialist, and part-time chaplain, with support from the hospital’s rehabilitation services. The mission of the team is diverse, including aiding in establishing goals of care, pain, and symptom management, spiritual guidance, and end-of-life care if needed. Similar to another recent retrospective study in children with oncologic diagnoses, no relationship was noted between the availability of palliative care consultation and rates of death at home [15]. The lack of impact on home death may be secondary to either long-term care planning being discussed at pediatric oncologic appointments as an outpatient, DNR status discussions taking place outside of inpatient admission prior to death, or outpatient arrangements for home hospice; these factors were not captured in our study.

The results of our study contrast two recent studies exploring patterns in end-of-life care in patients with oncologic diagnoses. A large study by Wolfe et al. [16] demonstrated a significant decrease in the number of children with oncologic diagnosis who died in the intensive care unit following the initiation of a pediatric advanced care team. They also noted earlier and more frequent discussions of hospice care. A separate study from the same institution by Ullrich et al. [17] demonstrated that following the initiation of a pediatric palliative care service, children were more likely to die outside of the ICU and receive less intervention-focused care in the 24 h prior to death. Our study results likely contrast these findings due to a low percentage of Oncology patients in our overall dataset (25%) compared with Oncology-only studies. Our study was not powerful enough to examine outcomes for Oncology patients alone.

We were able to identify the patient’s location, primary team, and physician obtaining the DNR in our study population. Following the introduction of a palliative care program, consultation rates grew to greater than 70% for all study patients, and the DNR orders were most commonly placed by the palliative care team. The increasing role of palliative care consultants in end-of-life care is likely reflected by time-demands of the primary service, increased provider comfort with end-of-life discussions for the consultant, and the nature of the multidisciplinary team, including social work and advanced practice nursing.

Previous studies have demonstrated a large range in the rates of DNR order placement prior to death in pediatric populations. One recent literature review found that death occurred following withdrawal and limitation of life-sustaining treatment in 30–65% of North American children’s hospitals [18]. The introduction of a multidisciplinary palliative care team significantly increased the rates of DNR order placement, from 30.8% to 39.2%, during our study period. The increase in DNR order placement is likely secondary to frequent palliative care consultation (>75% deceased patients), improved family awareness of prognosis and disease process, and the facilitation of communication.

The retrospective nature of this study provided significant limitations. We were limited by the data available in the electronic medical record at the time of the study. Many patients in the study lacked specific demographic information; most frequently, the variables of religion and primary language were omitted or not disclosed. Specific studies have demonstrated variation dependent upon familial income, educational level, or community structure, which were not available in our study [9]. Although they are the minority, only children with an inpatient DNR order placement were encompassed by our study. Those children with an outpatient DNR order or paper-based documentation were not included in this study.

The strengths of this study include the high volume of patients seen during the study period at our institution, which includes a high-volume emergency department, level-I trauma center, solid and bone marrow organ transplant teams, NICU, PICU, and CICU. The introduction of the pediatric palliative care team also provided an avenue for unique research during the study period. Further research is needed to elucidate the factors surrounding DNR order placement and end-of-life care in pediatrics. Targeted surveys and an analysis of the barriers to home discharge prior to death in the pediatric intensive care unit would likely have significant impacts.

## 5. Conclusions

Our study presents a large retrospective analysis of pediatric limitations of care and timing of death. The results demonstrate a large variation in the timing of death following DNR order placement when comparing the intensive care units and the general pediatric floor. No differences in the timing of death were noted between children of varying sex, ethnicity, and religion, where this information was available. The study demonstrates a marked increase in DNR rates following the initiation of a multidisciplinary palliative care team, but no change was detected in the interval between DNR placement and death. Further studies are needed to analyze physician attitudes, family beliefs, and barriers affecting end-of-life care in pediatrics.

## Figures and Tables

**Table 1:** Baseline patient characteristics and demographics.

Demographics	Value (*n* = 167)
Average age	6.54 years (3 days to 20 years)
Sex *^a^*	76 Females (45.5%)
	91 Males (54.5%)
Self-identified race *^a^*	92 Caucasian (55.1%)
	31 Black or African American (18.6%)
	6 Asian (3.5%)
	5 Hispanic/Latino/Spanish origin (3.0%)
	33 Not identified (19.8%)
Language *^a^*	87 English (52%)
	15 Spanish (9%)
	1 Vietnamese (0.5%)
	64 Not recorded (38%)
Primary diagnosis	29 Congenital heart disease (17%)
	33 Genetic syndrome (20%)
	25 Infectious (15%)
	28 Neuromuscular disease (17%)
	41 Oncologic diagnosis (25%)
	11 Trauma/arrest (6.5%)

*^a^* No significant difference (*p* > 0.05) in do-not-resuscitate (DNR) intervals to death between patients of differing sex, race, or primary language spoken.

**Table 2:** Palliative care consultation.

Name	Value
**Percent of patients with DNR**	
Total	33.3% (*n* = 167/471)
Pre-palliative care	30.8% (*n* = 66/214)
Post-palliative care	39.2% (*n* = 101/257); *p* = 0.05
**Interval between DNR and Death**	
Total	37.0 days
Pre-palliative care	27.0 days
Post-palliative care	43.1 days; *p* = 0.28
Likelihood of obtaining palliative care consultation	80% (*n* = 81/101)

Palliative care consultation was associated with a ~10% increase in DNR placement (*p* = 0.05), but no change in the interval between DNR placement and death (*p* = 0.28).
